# Low-dose paclitaxel modulates tumour fibrosis in gastric cancer

**DOI:** 10.3892/ijo.2013.1801

**Published:** 2013-01-29

**Authors:** TOMOYA TSUKADA, SACHIO FUSHIDA, SHINICHI HARADA, SHIROH TERAI, YASUMICHI YAGI, JUN KINOSHITA, KATSUNOBU OYAMA, HIDEHIRO TAJIMA, ITASU NINOMIYA, TAKASHI FUJIMURA, TETSUO OHTA

**Affiliations:** 1 Department of Gastroenterologic Surgery, Division of Cancer Medicine, Graduate School of Medical Science, Kanazawa University, Kanazawa, Ishikawa 920-8641, Japan; 2 Center for Biomedical Research and Education, School of Medicine, Kanazawa University, Kanazawa, Ishikawa 920-8641, Japan

**Keywords:** gastric cancer, human peritoneal mesothelial cell, paclitaxel, epithelial-mesenchymal transitions, fibrosis

## Abstract

Various treatments have been used for peritoneal dissemination, which is the most common mode of metastasis in gastric cancer, but sufficiently good clinical outcomes have not yet been obtained because of the presence of rich fibrous components and acquired drug resistance. Epithelialmesenchymal transition (EMT) is one of the major causes of tissue fibrosis and transforming growth factor-β (TGF-β) has a pivotal function in the progression of EMT. Smad proteins play an important role in the TGF-β signalling pathway. The TGF-β/Smad signalling pathway can be modulated by stabilising microtubules with paclitaxel (PTX). Here, we investigated whether paclitaxel can modulate TGF-β/Smad signalling in human peritoneal methothelial cells (HPMCs). To determine the cytostatic concentrations of antineoplastic agents in HPMCs, a 3-(4,5-dimethylthiazol-2-yl)-2,5-diphenyltetrazolium bromide (MTT) assay was performed using PTX, 5-fluorouracil and cisplatin. The minimum concentration that caused significant inhibition of TGF-β1-induced morphological changes in human peritoneal methothelial cells on pre-treatment with PTX was 5 nM at 48 h (cell viability: 87.1±1.5%, P<0.01). The TGF-β signalling cascade and the status of various fibrous components were evaluated by immunofluorescence staining, real-time quantitative PCR and western blotting. TGF-β signalling induced morphological changes, α-SMA expression and collagen I synthesis in HPMCs and PTX treatment suppressed these EMT-like changes. Moreover, PTX treatment markedly suppressed Smad2 phosphorylation. These data suggest that at a low-dose, PTX can significantly suppress the TGF-β/Smad signalling pathway by inhibiting Smad2 phosphorylation in the human peritoneum and that this can reduce stromal fibrosis.

## Introduction

Peritoneal dissemination is the most common mode of metastasis in gastric cancer. Various approaches have been assessed for the treatment of peritoneal dissemination, including systemic chemotherapy, intraperitoneal chemotherapy (1), extensive intraoperative peritoneal lavage (2) and aggressive surgery (3). The clinical outcome in gastric cancer patients with peritoneal dissemination has improved, but sufficiently good outcomes have not yet been obtained (4,5). In particular, acquired drug resistance and invasive scirrhous cell dissemination, characterized by rich fibrous components, are typical manifestations of clinical gastrointestinal disorders (e.g., ileus, obstructive jaundice and hydronephrosis). In cancer peritoneal dissemination, human peritoneal mesothelial cells (HPMCs), which are classified as epithelium in the broadest sense of the term, serve as a protective anatomical barrier and play an important role in the immunological response to infection and wound healing; they also contribute to cancer cell growth and fibrosis via epithelial-mesenchymal transition (EMT) (6,7).

EMT is an essential mechanism that guides proper development during several phases of embryogenesis (8). It is also related to pathological changes such as organ fibrosis (9) and tumour metastasis (10). EMT is characterised by loss of cell-cell adhesion and apical-basal polarity, followed by a shift in cytoskeletal dynamics toward front end-back end polarity and cell migration (11).

Transforming growth factor-β (TGF-β), the prototype member of a mammalian superfamily of growth factors that includes activins, bone morphogenetic proteins (BMPs), inhibins and nodal, is a common initiator of EMT. TGF-β and related factors evoke pleiotropic cellular responses through binding to transmembrane serine-threonine kinase receptor type I (TβR-I) and TβR type II (TβR-II) (12). The activated TGF-β receptors stimulate the phosphorylation of receptor-regulated Smad2 and Smad3 proteins (R-Smad), which in turn form complexes with Smad4 (co-Smad). This complex translocates from the cytoplasm into the nucleus, where the Smads regulate the transcription of target genes. The activity of inhibitory Smad7 (I-Smad) is the opposite of that exhibited by R-Smads; I-Smad downregulates TGF-β signalling (13). Some studies have previously shown that endogenous Smad2, Smad3 and Smad4 bind to microtubules in several cell lines and that this binding provides a negative regulatory mechanism to modulate TGF-β activity (14).

Paclitaxel (PTX), derived from the bark of the Pacific yew, *Taxus brevifolia*, is an antineoplastic agent that stabilizes polymerized microtubules and enhances microtubule assembly and thus arrests the cell cycle in the G0/G1 (low dose) and G2/M (high dose) phases, leading to cell death (15,16). PTX has been used in the treatment of peritoneal dissemination of ovarian and gastric cancers (17,18). Clinically, PTX has also been reported to improve intestinal stenosis due to fibrosis associated with the progression of peritoneal dissemination (19). At low concentrations, PTX has antiproliferative and antimigratory effects in vascular smooth muscle and endothelial cells (20,21) and has been widely applied clinically, e.g., in drug-eluting stents (22,23). Importantly, PTX has been shown to modulate TGF-β signalling, interrupting fibrosis in a murine systemic sclerosis model and in gallbladder myofibroblasts (24,25).

The aim of the present study was to evaluate the inhibitory effects of TGF-β-induced EMT in HPMCs at the cytostatic concentration of the antineoplastic agents PTX, 5-fluorouracil (5-FU) and cisplatin (CDDP).

## Materials and methods

### Antineoplastic agents

5-FU and CDDP were purchased from Sigma-Aldrich Inc. (USA). PTX was kindly provided by the Bristol-Myers Squibb Co. (Japan) and reconstituted in distilled water at appropriate concentrations and stored at −20°C until use.

### Cell lines and cell culture

HPMCs were isolated from surgical specimens of the human omentum, as previously described (26). Omental specimens were obtained, with informed consent, from patients undergoing elective abdominal surgery. Tissue samples were collected in ice-cold phosphate buffered saline (PBS) to minimize cell degeneration. Contaminating red blood cells were removed by extensive PBS washes and samples were incubated in pre-warmed PBS containing 0.125% trypsin/EDTA (Gibco/Invitrogen, USA) for 30 min at 37°C. The suspension was passed through a 100-*μ*m-pore nylon mesh (Becton-Dickinson, Japan) to remove undigested fragments and was then centrifuged at 1,500 rpm for 5 min. Dissociated cells were cultured in RPMI-1640 medium (Gibco/Invitrogen) supplemented with 10% heat-inactivated fetal bovine serum (Nichirei Bioscience Inc., Japan), 100 IU/ml penicillin, 100 mg/ml streptomycin (Gibco/Invitrogen) and 2 mM glutamine (Nissui Pharmaceutical Co. Ltd., Japan). The cells were seeded in gelatin-coated 75-cm^2^ flasks (BD BioCoat, USA) and cultured in 10 ml of medium at 37°C in a humidified atmosphere of 5% CO_2_ in air.

Human gastric cancer cell lines (MKN45) were obtained from the American Type Culture Collection (USA). MKN45 cells were cultured in the media indicated above for HPMC. The cells were grown to confluence and were harvested by trypsinisation with 0.25% trypsin/EDTA. Confluent HPMCs were trypsinised with 0.125% trypsin/EDTA before use. HPMCs were then transferred to serum-free medium for 24 h, after which they were continuously exposed to 5 ng/ml of recombinant human TGF-β1 (Sigma-Aldrich, Inc., USA) for 48 h. Finally, they were transferred to RPMI-1640 containing 10% FBS, which caused a shift in the morphology of the cells, resulting in activated HPMCs (a-HPMCs). HPMCs were used from passage 1 to 3 in all experiments.

### Cell growth assay

The viability of HPMC and MKN45 cells treated with antineoplastic agents was determined by a standard 3-(4,5-dimethylthiazol-2-yl)-2, 5-diphenyltetrazolium bromide (MTT) assay. MKN45 cells were seeded at 5×10^3^ per well in 96-well plates and were incubated overnight at 37°C. HPMC cells were seeded at 5×10^3^ per well on gelatin-coated 96-well microplates (BD BioCoat). After incubation, the supernatant was discarded and replaced with fresh serum-free medium. Antineoplastic agents were dissolved in PBS and added to the cell culture medium at various concentrations (5-FU, 0-10 *μ*M; CDDP, 0–10 *μ*M; PTX, 0–100 nM). At 48 h after exposure to antineoplastic agents, the supernatant was discarded and MTT solution was added to each well (final concentration, 500 *μ*g/ml) and incubated at 37°C for 3 h. Then, the supernatant was removed and 150 *μ*l of dimetylsulphoxide (DMSO; Wako, Japan) was added. The absorbance of the solution was read at 540 nM with a microplate reader (Bio-Rad 550; Bio-Rad, Japan). The percentage inhibition was determined by comparing the cell density of the drug-treated cells with that of untreated controls. All the experiments were repeated a minimum of 3 times.

### Histology and immunofluorescence

Subconfluent HPMCs were transferred to serum-free medium for 24 h, after which they were continuously exposed to one of the following treatments: 0.5 or 1 *μ*M of 5-FU, 5 or 10 *μ*M of CDDP and 1 or 5 nM of PTX for 1 h. Subsequently, 5 ng/ml of recombinant human TGF-β1 was added. After 48-h exposure to TGF-β1, morphological changes in HPMCs were observed by phase-contrast microscopy. Cells were then harvested for immunostaining.

For visualizing E-cadherin and α-SMA in HPMCs, the cells were grown on 4-well collagen type I-coated culture slides (BD BioCoat) and then fixed in a mixture of methanol and acetone (1:1) for 15 min. Immunostaining was performed as reported earlier (27). Briefly, the slides were immersed in methanol containing 0.3% H_2_O_2_ for 30 min, blocked with 3.3% normal goat serum in PBS and incubated with the anti-E-cadherin antibody (H-108, rabbit polyclonal IgG, diluted 1:100; Santa Cruz Biotechnology, Inc. USA) and anti-α-SMA (1A4, mouse monoclonal IgG, diluted 1:100; DakoCytomation, Denmark) at 4°C overnight. Following the PBS washes, immunoreactivity was visualized by incubating the sections with anti-mouse IgG antibody conjugated with Alexa Fluor^®^ 488 and anti-rabbit IgG antibody conjugated with Alexa Fluor^®^ 546 (Molecular Probes/Invitrogen, USA) (1:400) for 1 h at room temperature. The slides were observed with an immunofluorescence microscope (BX50/BX-FLA; Olympus, Japan).

### Western blotting

Immunoblot analysis was performed as described previously (28). The cells were lysed in RIPA buffer [50 mmol/l Tris-HCl (pH 8.0), 150 mmol/l sodium chloride, 0.5 w/v% sodium deoxycholate, 0.1 w/v% sodium dodecyl sulphate, 1.0 w/v% NP-40 substitute (Wako, Japan)] containing 1% protease inhibitor cocktail (Sigma-Aldrich, Inc.). The protein concentration of each sample was measured using a BCA protein assay kit (Pierce Biotechnology, USA). Whole-cell lysates were prepared in denaturing SDS sample buffer and subjected to SDS-PAGE (Atto Co. Ltd., Japan). Proteins were transferred to PVDF membranes (Bio-Rad, USA) and were then blocked with commercial gradient buffer (EzBlock, Atto Corp.) at room temperature for 30 min. The immunoblots were visualized using an ECL Plus kit (GE Healthcare Japan Ltd., Japan). The antibody-antigen complex was detected using an ECL western blotting detection kit (GE Healthcare Japan Ltd.) and the Light-Capture system (Atto). We used the following primary antibodies: anti-E-cadherin (H-108, rabbit polyclonal IgG, diluted 1:1,000; Santa Cruz Biotechnology, Inc.), anti-α-SMA (1A4, mouse monoclonal IgG, diluted 1:5,000; DakoCytomation), anti-Smad2/3 (E-20, goat polyclonal IgG, diluted 1:1,000; Santa Cruz Biotechnology, Inc.) and anti-β-actin (AC-15, mouse monoclonal IgG, diluted 1:10,000; Sigma).

### Relative quantification by real-time quantitative PCR

Total RNA was extracted from HPMCs with an RNeasy mini kit (Qiagen, USA) and treated with an RNase-free DNase set (Qiagen), following the manufacturer’s recommendations. An Agilent 2100 Bioanalyzer microfluidic assay (Agilent Technologies, USA) was used to assess RNA integrity. Spectrophotometric and fluorometric methods were combined to quantitate RNA. cDNA was generated from RNA using a reverse transcription kit (Applied Biosystems, USA). Total RNA (1 *μ*g) was reverse transcribed in a total volume of 20 *μ*l by using 100 U of reverse transcriptase, 2.0 *μ*l 10X RT buffer, 2.0 *μ*l 10X random primers and 1.0 *μ*l of 20 U/*μ*l RNase inhibitor. The mixture was incubated for 10 min at 25°C, 120 min at 37°C and 5 min at 85°C; it was then rapidly cooled on ice. The cDNA samples were stored at −20°C.

Real-time qPCR was performed on an M×3005P Multiplex Quantificative PCR system with the MxPro QPCR software (Stratagene, USA). TaqMan^®^ Universal Master Mix (Applied Biosystems) was used for PCR. In a final volume of 20 *μ*l, 1 *μ*l of cDNA was amplified using the following TaqMan^®^ assays (Applied Biosystems): Smad2 (Hs00998181_gH), collagen type I (Hs01076775_g1), GAPDH control reagents and β-actin control reagents. The PCR cycling conditions were as follows: 50°C for 2 min; 95°C for 10 min; and 40 cycles of 95°C for 15 sec and 60°C for 1 min. All qPCR reactions were performed in triplicate. The threshold cycle (Ct) method was used for quantification. For relative quantification, Smad2 and collagen type I mRNA levels, normalized to endogenous house-keeping controls (GAPDH and β-actin), were divided by normal control sample values (normal HPMC samples) to generate the relative quantification to calibrator (rel. quant. to cal.). All the experiments were repeated a minimum of 3 times.

### Quantitation of phosphorylated Smad2

To determine whether the modulation of TGF-β1 transcriptional activity by PTX correlated with a change in the phosphorylation state of Smad2, we performed western blot analysis. Subconfluent HPMCs were prepared as mentioned earlier; 1 or 5 nM of PTX was added for 1 h, followed by exposure to 5 ng/ml of recombinant human TGF-β1 and western blotting was performed using an anti-phospho-Smad2 antibody (Ser 465/467, A5S, rabbit polyclonal IgG, diluted 1:1,000; Millipore, USA). The antibody-antigen complex was detected using a Light-Capture system (Atto) and was then quantified using the CS analyzer program (Atto). All the experiments were repeated 3 times.

### Statistical analysis

Values are expressed as means ± standard error (SE). One-way analysis of variance (ANOVA) was performed using SPSS 10.0 (SPSS Inc., USA). Significance was defined as P<0.05.

## Results

### Determination of minimum cytostatic concentration of anti-neoplastic agents

**Figure 1:**
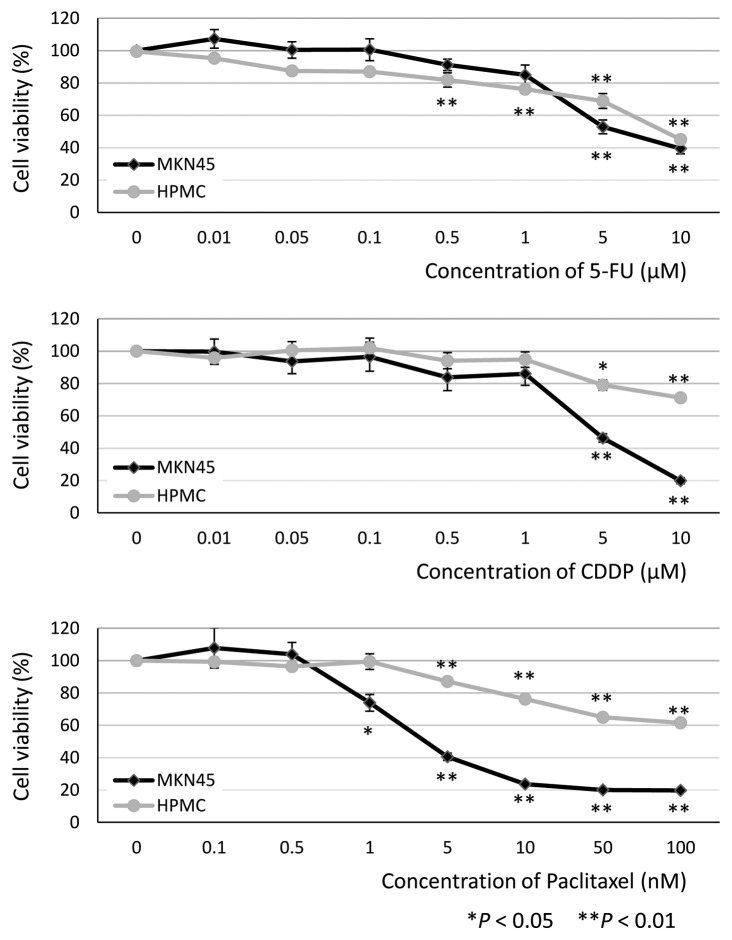
Antiproliferative effects of antineoplastic agents in HPMCs and MKN45 cells. Cell viability was assessed after 48-h exposure to a single-dose of 5-FU (0-10 *μ*M), CDDP (0–10 *μ*M) and PTX (0–100 nM) in serum-free medium. The results are provided in terms of means ± SE values of 3 different experiments.

MTT assays were performed in HPMC and MKN45 cells (as a comparator) to determine the minimum cytostatic concentrations of the antineoplastic agents. The concentrations required for significant inhibition of HPMC viability at 48 h were 0.5 *μ*M 5-FU (81.9±4.4%, P<0.01, n=9), 5 *μ*M CDDP (79.0±3.2%, P=0.032, n=9) and 5 nM PTX (87.1±1.5%, P<0.01, n=9) (Fig. 1). On the basis of these results, we decided to use the following concentrations for subsequent experiments: 0.5 and 1 *μ*M 5-FU, 5 and 10 *μ*M CDDP and 1, 5 and 10 nM PTX.

### Effect of PTX on morphological changes, and E-cadherin and α-SMA expression in HPMCs

**Figure 2:**
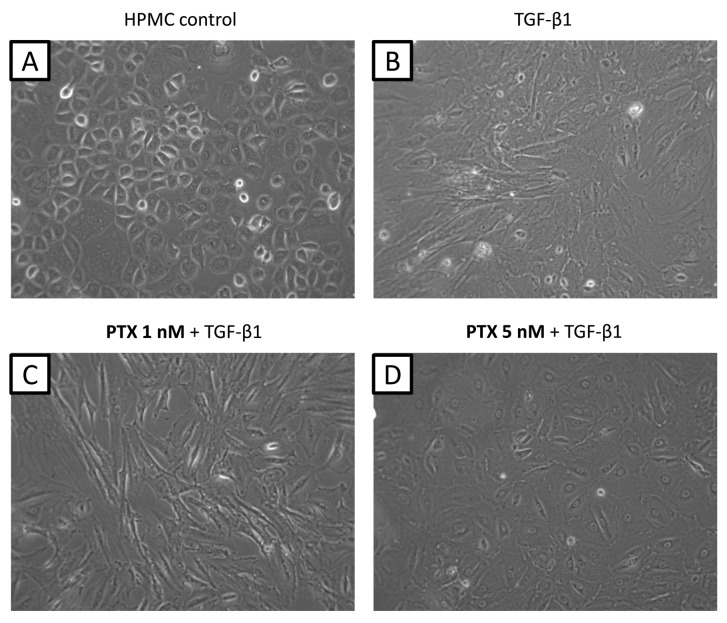
Effect of PTX on the morphological changes in HPMCs. (A) The control mesothelial cells exhibited a cobblestone-like growth pattern. (B) After continuous TGF-β1 exposure for 48 h, the HPMC morphology converted to a spindle fibroblast-like morphology. (C) Pre-treatment with 1 nM PTX did not affect the cell morphology induced by TGF-β1. (D) Pre-treatment with 5 nM PTX resulted in rounding of the cells. Original magnification ×200.

Morphological changes were observed in cultured HPMCs after adding TGF-β1 (5 ng/ml) for 48 h. Control HPMCs without TGF-β1 treatment showed an epithelial morphology with a cobblestone appearance (Fig. 2A); however, treated HPMCs converted to a morphology with a spindle fibroblastic pattern (Fig. 2B). Pre-treatment with 1 nM PTX did not suppress the morphological changes induced by TGF-β1 (Fig. 2C). However, cells pre-treated with 5 nM PTX appeared rounded in shape suggesting that at this dose, PTX had an inhibitory effect on the action of TGF-β1 (Fig. 2D). This inhibition of morphological changes was not observed with other antineoplastic agents (data not shown).

**Figure 3:**
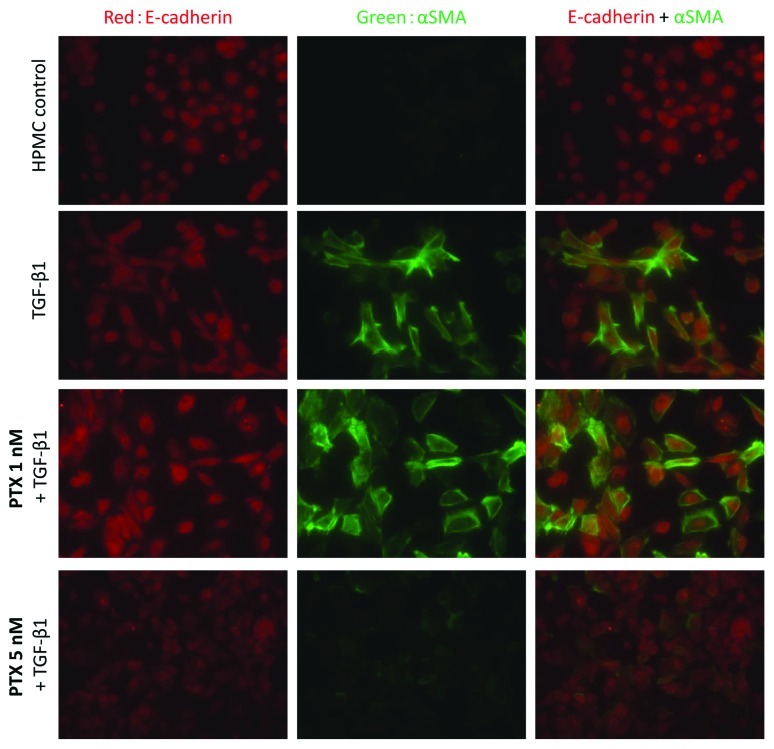
Immunofluorescence examination of E-cadherin and α-SMA expression in HPMCs. Representative photomicrographs of HPMC, which were labelled with antibodies to E-cadherin (red) and α-SMA (green). TGF-β1 induced increase in the expression of α-SMA and 5 nM PTX suppressed α-SMA expression. Original magnification ×400.

**Figure 4:**
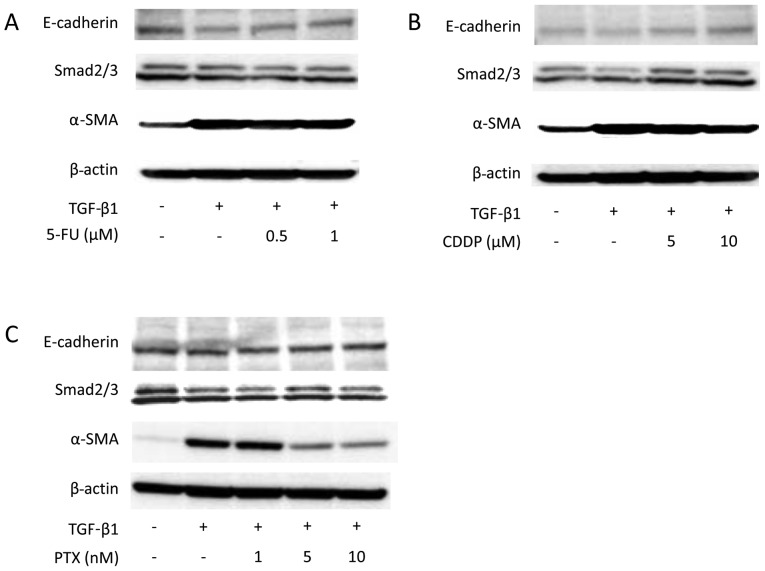
Western blot analysis of E-cadherin (120 kDa), Smad2/3 (55 kDa) and α-SMA (42 kDa). (A) α-SMA expression was higher in a-HPMCs than in HPMCs and was suppressed by pre-treatment with 5 nM PTX. (B and C) It was not clear whether the other antineoplastic agents affected α-SMA expression. Total expression of Smad2/3 was not affected by 48-h exposure to TGF-β1 in any of the experiments.

Immunofluorescence analysis of the morphological changes showed that α-SMA expression increased on TGF-β1 treatment and was suppressed by pre-treatment with 5 nM PTX (Fig. 3). Western blot analysis also showed that α-SMA expression increased on TGF-β1 stimulation. Suppression of α-SMA expression was not detected with either 5-FU or CDDP treatment (Fig. 4); however, pre-treatment with 5 and 10 nM PTX did suppress TGF-β1-induced α-SMA expression.

### Effect of antineoplastic agents on collagen production and TGF/Smad signalling in HPMC

**Figure 5:**
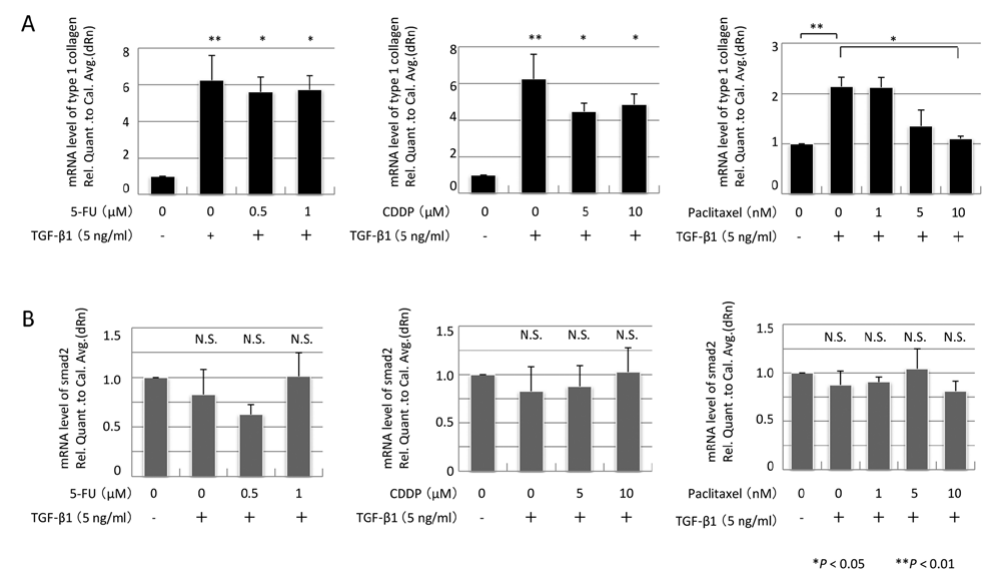
Relative quantification by real-time qPCR for collagen type I and Smad2 mRNA expression. For relative quantification, the collagen type I and Smad2 mRNA levels, normalized to the internal controls (GAPDH and β-actin), were divided by 1 normalized control sample value (calibrator sample) to generate the relative quantification to the calibrator (rel. quant. to cal.). (A) The collagen type I mRNA expression level was higher in a-HPMCs than HPMCs and was suppressed by 10 nM PTX pre-treatment; however, the other antineoplastic agents had no effect. (B) The Smad2 mRNA expression level was assessed and no significant differences were observed in any experiments. The results have been expressed as means ± SE (n=3).

Collagen I mRNA expression was assessed by real-time qPCR. TGF-β1 stimulation induced parallel increases in both α-SMA and collagen I mRNA expression (Fig. 5A). Pre-treatment with 5-FU or CDDP did not suppress collagen I mRNA expression; however, pre-treatment with 10 nM PTX promoted attenuation of collagen I mRNA expression (P=0.031, n=3).

**Figure 6:**
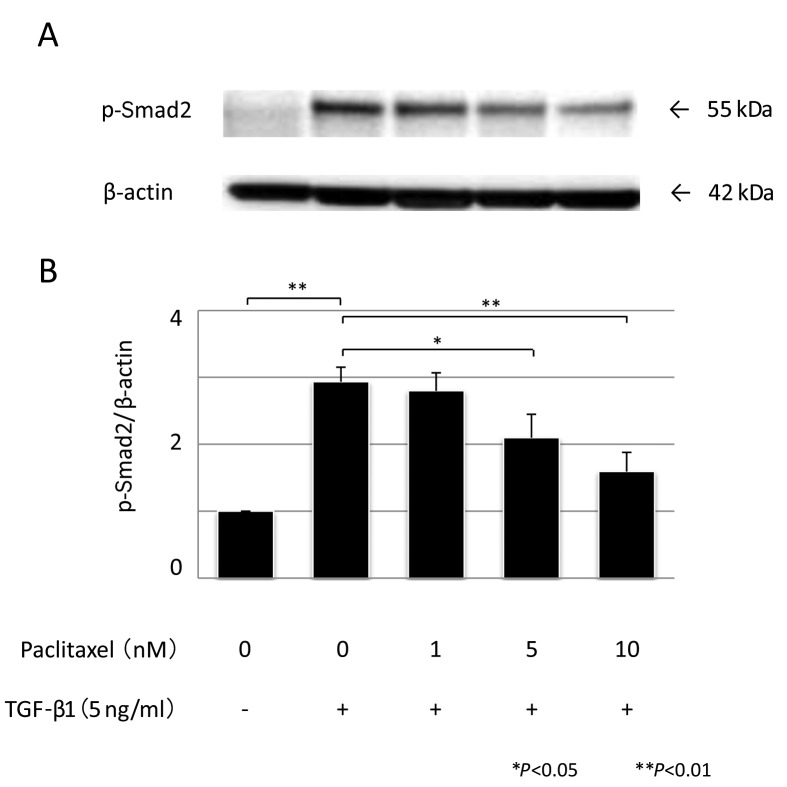
Effect of PTX on the phosphorylation of Smad2 in HPMCs. (A) Western blot analysis for phosphorylated Smad2 (55 kDa). Phosphorylation of Smad2 was detected after 1-h exposure to TGF-β1, while pre-treatment at 5 or 10 nM PTX inhibited phosphorylation. (B) Densitometric analyses were performed from 3 independent experiments; data are expressed as means ± SE.

To investigate the TGF/Smad pathway, Smad2/3 and Smad2 mRNA expression were assessed, but there were no alterations in their expression (Figs. 4 and 5B). In contrast, TGF-β1 induced a fourfold increase in Smad2 phosphorylation (P<0.01, n=3). Pre-treatment with either 5 or 10 nM PTX significantly suppressed TGF-β1-induced phosphorylation of Smad2 (P=0.042 and P <0.01, respectively, n=3) (Fig. 6).

## Discussion

In the present study, we investigated the minimum cytostatic concentration of PTX that would not cause cytotoxicity in HPMCs. We had previously reported that TGF-β1-mediated activation of HPMCs is one of the origins of cancer-associated fibroblasts and can promote peritoneal fibrosis (6). Low-dose PTX might have the potential to reduce TGF-β1-mediated activation of HPMCs in the peritoneal microenvironment.

The diffusely infiltrating human scirrhous gastric carcinoma is characterized by cancer cell infiltration and proliferation accompanied with extensive stromal fibrosis (29). At the peritoneal dissemination site, cancer cells usually generate a supportive microenvironment by producing stroma-modulating growth factors such as the fibroblast growth factor (FGF) family, platelet-derived growth factor (PDGF), epidermal growth factor (EGF) ligands, vascular endothelial growth factor (VEGF) family, interleukins and TGF-β (30,31). In particular, TGF-β1 expression is correlated with the malignant potential of scirrhous gastric cancer (32) and contributes to adhesion, migration and invasion in the peritoneal dissemination of scirrhous gastric cancer (33). In addition, TGF-β produced by orthotopic fibroblasts has been shown to contribute to cell growth and extensive stromal fibrosis at the primary cancer site (34) and stimulates both the invasion and adhesion of scirrhous gastric cancer cells to the peritoneum (35,36).

In Japanese populations, 3-h infusions of PTX at the clinical dosages of 105, 135, 180, 210, 240 and 270 mg/m^2^ have been shown to result in peak plasma concentrations of 2600–14,000 nM. Peak plasma levels ranging from 40 to 120 nM have been obtained on administering PTX over 24 h (37). However, inhibition of TGF-β/Smad signalling can be achieved with very low doses of PTX. In addition, inhibition of migration and proliferation potential by different concentrations of PTX has been observed in tumour cell lines (38), epithelial cells (39), fibroblasts (40,41) and vascular smooth muscle cells (21). However, no studies have investigated the effect of PTX on HPMCs and no guidelines are available regarding the appropriate concentration for inhibiting fibrosis.

We found that the minimum cytostatic concentration of PTX was 5 nM. Interestingly, in the gastric cancer cell line, MKN45, exposure to 5 nM PTX resulted in lesser cell viability than the 50% inhibitory concentration (IC_50_), suggesting that the cytostatic concentration of PTX for HPMCs might be cytotoxic to gastric cancer cells. We have also verified that TGF-β1 induces a morphological change in HPMC and an associated elevation in α-SMA expression. These fibroblastic changes in HPMCs contribute to fibrosis by inducing collagen synthesis. Low-dose PTX (5 nM) inhibited a series of changes associated with EMT.

The Smad pathway plays a major role in the EMT process. R-Smads (Smad2 and Smad3) are composed of 3 regions: the N-terminal Mad-homology (MH) 1 domain that has DNA-binding activity, the C-terminal MH2 domain that has protein-binding properties and a middle linker region (42). The C-terminal phosphorylation of R-Smads is mediated by the activated TβR-I receptor, whereas middle linker region phosphorylation is mediated by mitogen-activated protein kinase (MAPK) (13). Other recent studies have shown that TGF-β can also activate non-Smad signalling cascades, including the MAPK pathway, leading to activation of MAPK-Erk, Jun N-terminal kinase (JNK) and p38MAPK (43,44). In rat peritoneal mesothelial cells, the JNK-Smad3 pathway contributes to peritoneal fibrosis (45), but the phosphorylation of R-Smads at the middle linker regions has been suggested to be cell specific (46). Therefore, we focused on the Smad pathway, which is common to many cells and on Smad2 phosphorylation, instead of Smad3 in HPMCs.

In the present study, we showed that TGF-β1 stimulation resulted in the phosphorylation of Smad2 in HPMCs and that this phosphorylation was inhibited by pre-treatment with 5 nM. In contrast to our findings, Wendling *et al* reported that 5-FU blocked TGF-β actions in human fibroblasts although this was in a different cell line and with a different concentration of 5-FU (47). We also determined that CDDP did not alter the action of TGF-β in HPMCs.

In conclusion, we have shown that low-dose PTX can significantly suppress TGF-β/Smad signalling by inhibiting Smad2 phosphorylation and decrease stromal fibrosis in human peritoneum cells. While our study does not prove that this happens *in vivo*, low-dose PTX has been shown to attenuate fibrosis in a rat model of unilateral ureteral obstruction (48). Furthermore, low-dose PTX (5 nM) prevented peritoneal fibrosis and was cytotoxic to gastric cancer cells. Therefore, combination therapies with low-dose cytostatic PTX and other cytotoxic antineoplastic agents could potentially become the expected regimen for peritoneal dissemination of gastric cancer. We hope that the results of the present study will provide an impetus for future investigations of novel treatment strategies for fibrotic peritoneal dissemination of gastric cancer.
